# Pembrolizumab for recurrent/metastatic head and neck squamous cell carcinoma in an Asian population

**DOI:** 10.1097/MD.0000000000009519

**Published:** 2017-12-29

**Authors:** Wen-Chun Chen, Pen-Yuan Chu, Yu-Ting Lee, Wen-Bin Lu, Chun-Yu Liu, Peter Mu-Hsin Chang, Muh-Hwa Yang

**Affiliations:** a Department of Medicine, Taipei Veterans General Hospital; b School of Medicine, National Yang-Ming University; c Department of Otolaryngology, Taipei Veterans General Hospital, Taipei; d Department of Medicine, Chiayi Branch, Taichung Veterans General Hospital, Chiayi, Taiwan; e Department of Medical Oncology, Wujin People's Hospital Affiliated to Jiangsu University, Jiangsu, China; f Division of Medical Oncology, Department of Oncology, Taipei Veterans General Hospital; g Institute of Clinical Medicine, National Yang-Ming University, Taipei, Taiwan.

**Keywords:** anti-PD1, head and neck squamous cell carcinoma, pembrolizumab, recurrent/metastatic

## Abstract

Head and neck squamous cell carcinoma (HNSCC) has a high prevalence and is a major cause of cancer deaths in Taiwan. However, there is still no effective salvage therapy that prolongs the life expectancy of patients with recurrent/metastatic (R/M) HNSCC. Immune checkpoint therapy that targets the programmed cell death protein 1 (PD-1) may provide clinical benefit for these patients. We analyzed 22 R/M HNSCC patients who received pembrolizumab, a monoclonal antibody against PD-1, as salvage therapy. Intravenous pembrolizumab was given at a fixed dosage of 100 or 200 mg every 3 weeks. Three patients also received local palliative radiotherapy, but no patients received chemotherapy or targeted drugs. Seventeen patients (77.3%) received at least 3 cycles of pembrolizumab. Based on Response Evaluation Criteria in Solid Tumors criteria (ver. 1.1), 2 patients (9.1%) had complete response, 5 (22.7%) had partial response, and 6 (27.3%) had stable disease, corresponding to a disease control rate of 59.1%. Four patients had confirmed disease progression, 2 of whom had continuous progression over the target lesion after shrinkage of other metastases. One patient developed immune-related pneumonitis that resolved quickly after steroid treatment. Another patient developed itchy skin rashes immediately after administration of pembrolizumab, and this was controlled by an antihistamine. There were no other severe adverse effects. Pembrolizumab is beneficial and well-tolerated for some patients with refractory R/M HNSCC. However, it is important to identify biomarkers to identify the most responsive patients when designing future trials.

## 1 Introduction

Head and neck squamous cell carcinoma (HNSCC) is the 6th most common cancer worldwide, and the 4th leading cause of cancer deaths among males in Taiwan.^[1]^ Patients with recurrent or metastatic (R/M) HNSCC have a poor prognosis, and there is no effective salvage therapy. Anti-epidermal growth factor receptor monoclonal antibodies, such as cetuximab, plus platinum-fluorouracil chemotherapy improve overall survival (OS), and these regimens are now standard first-line treatments for R/M HNSCC.^[1–3]^ Taxanes and methotrexate are usually used as second-line treatments. Recent studies have examined the effect of afatinib on patients with R/M HNSCC after failure of platinum-based therapy.^[4,5]^ However, these therapies provide limited benefit, and the associated toxicities may impair patient quality of life. A more effective and less toxic therapy is needed for these patients.

Pembrolizumab, a monoclonal antibody that targets the programmed cell death protein 1 (PD-1), was approved for the treatment of melanoma and R/M HNSCC in August 2016, and also has efficacy against some advanced solid tumors. A recent phase Ib trial of pembrolizumab for treatment of R/M HNSCC indicated the overall response was about 18%, and the median progression-free survival (PFS) was 2 months, suggesting that this treatment may benefit HNSCC patients who previously received intensive treatment.^[6]^ Another randomized, open-label, phase III trial of nivolumab, another monoclonal antibody that also binds to PD-1, reported the 1-year survival rate was approximately 36% in the nivolumab group but 16.6% in the control group (standard single-agent systemic therapy).^[7]^

In this pilot study, we enrolled R/M HNSCC patients who were treated with pembrolizumab to assess its antitumor effects, effect on survival, and safety in an Asian population.

## 2 Materials and methods

### 2.1 Patients

We retrospectively reviewed cases with R/M HNSCC who were treated with pembrolizumab at Taipei Veterans General Hospital between January and December 2016. All enrolled patients had pathologic confirmation of HNSCC, and were referrals from primary or secondary health professionals or newly diagnosed at Taipei Veterans General Hospital. All patients’ clinical characteristics, definitive treatments, therapeutic strategies (including sequential radiation therapy), toxicities, and comorbidities were obtained. All patients were followed up until death or the last follow-up date. This study was approved by Taipei Veterans General Hospital's Institutional Review Board (IRB No. 2016-09-009bc).

### 2.2 Treatment

All enrolled patients received intravenous pembrolizumab (100 or 200 mg) during a 30-min period every 3 weeks. This treatment continued until there were unacceptable grade 3 to 4 adverse effects, disease progression confirmed by imaging, a physician decided to discontinue treatment, or the patient withdrew consent. Pembrolizumab treatment could be continued in the presence of disease progression, as assessed clinically or radiographically, if the investigator determined it provided clinical benefit.

### 2.3 Response, outcomes, and statistical analysis

Descriptive statistics were used to summarize patients’ demographic and clinical characteristics, disease stage, treatment modality, and treatment-associated toxicities. Survival was calculated as the date of the first pembrolizumab treatment to the date of death or the last follow-up visit. PFS and OS were analyzed by Kaplan–Meier analysis. Progression was defined as development of distant metastases or local recurrence. Tumor size was calculated before pembrolizumab treatment and at after least 1 course of treatment, based on radiographic evidence every 3 months. The maximum change of target lesion size was evaluated based on Response Evaluation Criteria in Solid Tumors (RECIST) ver. 1.1. Treatment-related toxicities were evaluated by Common Terminology Criteria for Adverse Events. All statistical analysis was performed using IBM SPSS Statistics (version 22). A *P* value < .05 was defined as significant.

## 3 Results

### 3.1 Patient characteristics

Between January 2016 and December 2016, we identified 22 patients (5 women and 17 men) with R/M HNSCC who received immunotherapy with pembrolizumab (Table 1). The median age was 61 years (interquartile range [IQR]: 47–69 years). The primary sites of the HNSCC were the oral cavity (n = 9, 40.9%), oropharynx (n = 2, 9.1%), hypopharynx (n = 4, 18.2%), neck lymphadenopathy with unknown primary site (n = 4, 18.2%), and others (nasopharyngeal cancer [n = 2, 9.1%] and squamous cell carcinoma transformed from thyroid cancer [n = 1, 4.5%]). Three patients received combined local palliative radiation over the primary sites of recurrent tumors. No other chemotherapy or targeted drugs were given with pembrolizumab.

**Table 1:**
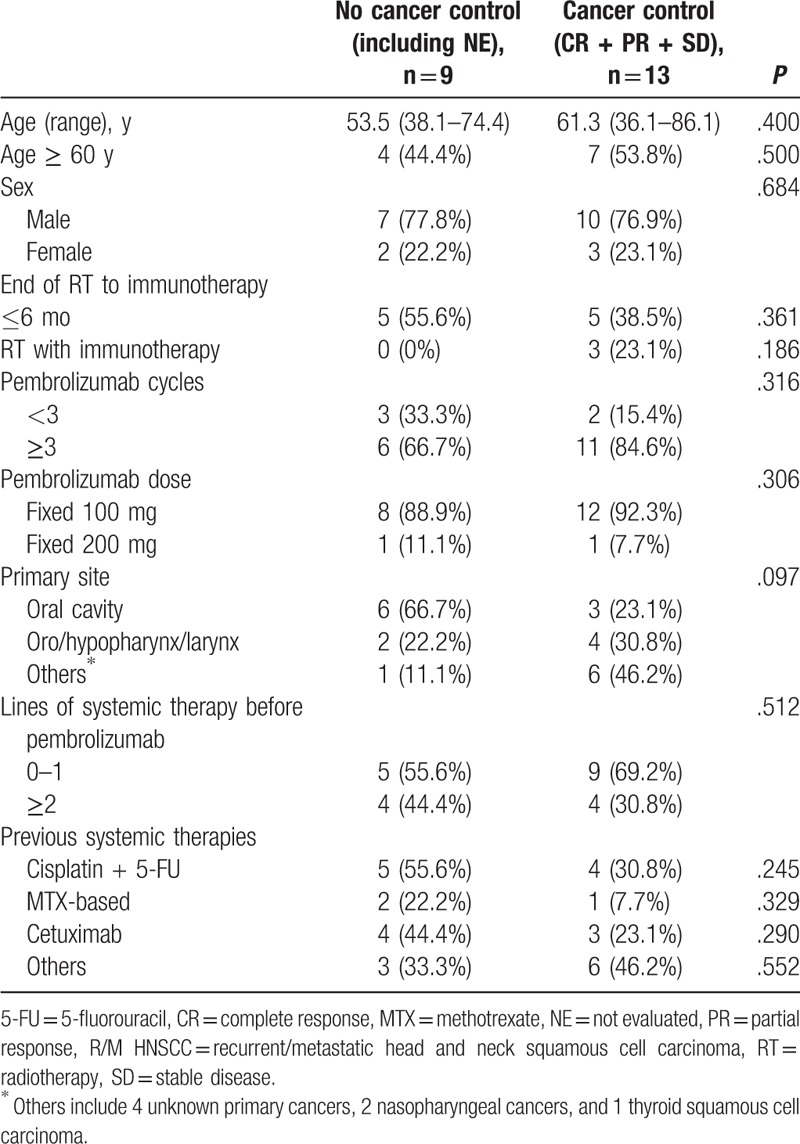
Baseline characteristics of patients with R/M HNSCC who received pembrolizumab and whose cancer was or was not controlled (n = 22).

We also evaluated the front-line treatments that patients received after initial confirmation of R/M HNSCC. Five patients (22.7%) received pembrolizumab as a first-line treatment after confirmation of R/M HNSCC due to poor performance status and general health status, early relapse in the 6 months after a previous cisplatin/5-FU treatment, or the patient's request to avoid chemotherapy. Nine (40.9%) patients received a first-line systemic therapy before pembrolizumab, and 8 (36.4%) received 2 or more systemic treatments before pembrolizumab. The systemic chemotherapy regimens given prior to pembrolizumab were cisplatin or carboplatin combined with 5-FU (n = 9, 40.9%), a methotrexate-based regimen (n = 3, 13.6%), cetuximab-based chemotherapy (n = 7, 31.8%), and others (n = 9, 40.9%). Human papillomavirus (HPV) status was retrospectively evaluated within these patients. Only 4 patients had p16 staining data, including 3 patients (1 oropharyngeal SCC, 1 nasopharyngeal SCC, and 1 hypopharyngeal SCC) with p16 negative and 1 patient (Patient No. 9, oropharyngeal SCC) with p16 positive. HPV polymerase chain reaction (PCR) examination for tumor tissue HPV had demonstrated undetectable HPV DNA in the patient with p16 staining positive. The correlation of HPV status with response rate cannot be assessed due to small number.

### 3.2 Outcome

**Figure 1:**
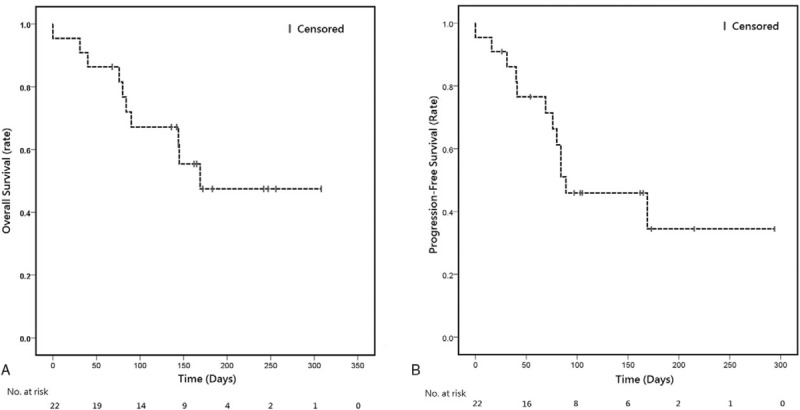
Kaplan–Meier survival curves for (A) progression-free survival and (B) overall survival of patients with recurrent/metastatic head and neck squamous cell carcinoma who were treated with pembrolizumab.

**Figure 2:**
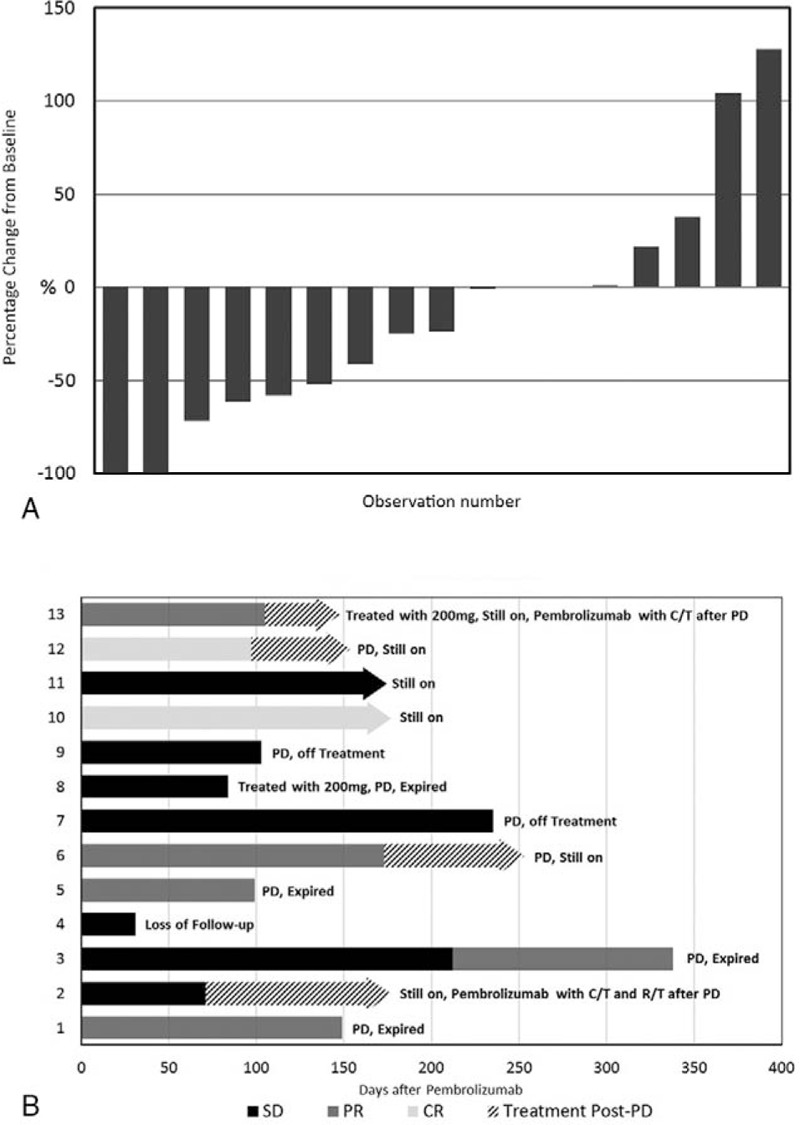
(A) Waterfall plot of tumor size in patients with measurable lesions and (B) Swimmer plot of treatment duration in evaluable patients. C/T = chemotherapy, CR = complete response, PD = progressive disease, PR = partial response, R/T = radiotherapy, SD = stable disease.

There were 17 evaluable patients (77.3%) who received at least 1 image follow-up after pembrolizumab treatment. The median PFS was 140 days, and the OS was 169 days (Fig. 1). Based on RECIST criteria (ver. 1.1), 2 patients (9.1%) had a complete response (CR), 5 patients (22.7%) had a partial response (PR), and 6 patients (27.3%) had stable disease, corresponding to a disease control rate (DCR) of 59.1%. Six patients who ever showed controlled disease under front-line systemic treatment are all responsive to pembrolizumab treatment (100%). Among the other 11 patients who failed to response to front-line systemic treatment, 6 (63%) showed controlled disease after pembrolizumab salvage treatment. Figure 2 shows a waterfall plot of tumor size and a swimmer plot of treatment duration for each of the 17 evaluable patients. We describe 4 notable patients below, 2 with complete remission, 1 with nonconcordant changes of metastatic lesions and target lesions, and another with immune-related pneumonitis.

**Figure 3:**
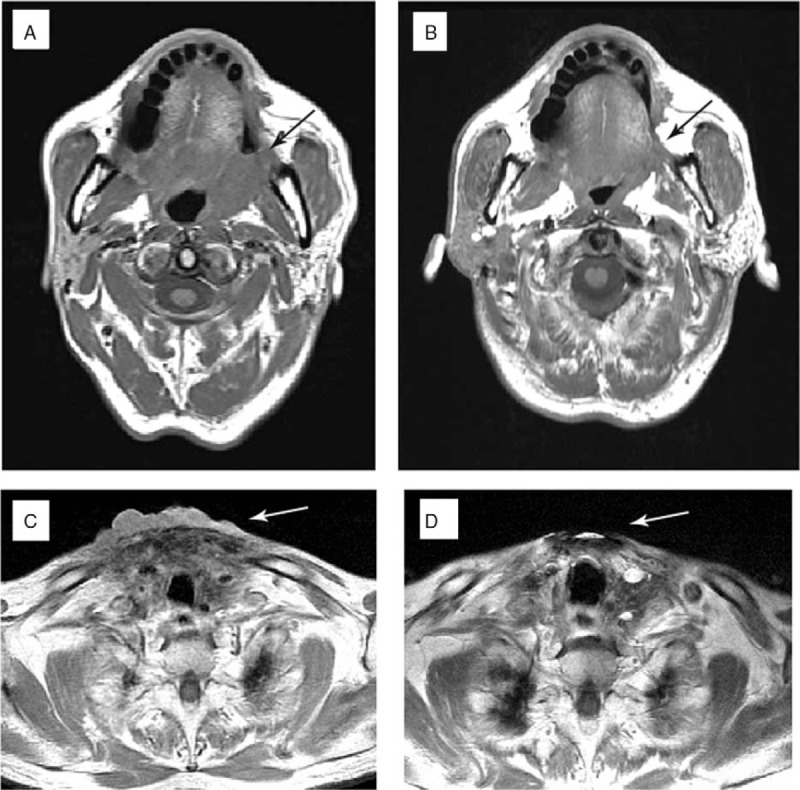
Patient No. 10 had a squamous cell carcinoma in the left hard palate, with tumor recurrence over the left palatine tonsil and invasion into the masseter muscle (A, black arrow). Complete response occurred after 6 months of treatment with pembrolizumab (B, black arrow). Patient No. 12 had a papillary thyroid cancer with surrounding invasion, and received palliative resection in 2014. Tumor recurrence (C, white arrow) in 2015 and a biopsy revealed squamous cell carcinoma, probably originating from the thyroid. A neck magnetic resonance imaging (D) showed complete response after 3 months of treatment with pembrolizumab.

Patient No. 10 was diagnosed with squamous cell carcinoma of the left hard palate, with an initial pathological stage of pT1N0M0 (Fig. 3A and B). He received transoral tumor excision in 2012, but recurrent lymphadenopathy appeared over the left neck in 2015. The patient received modified left neck radical lymph node dissection (January 15, 2015) with adjuvant radiotherapy. Tumor recurrence occurred at the left palatine tonsil, with invasion into the masseter muscle, and he was classified as pathological stage rT4aN0M0 and clinical stage IVA based on follow-up imaging. The patient then accepted cytotoxic chemotherapy (1 cycle) with cisplatin, fluorouracil, and docetaxel with weekly cetuximab in June 2016. Following the patient's request, he started pembrolizumab (fixed dose of 100 mg every 3 weeks) in July 2016. The last follow-up was in December 2016, at which time there was tumor shrinkage without local recurrence or metastasis.

Patient No. 12 was diagnosed with papillary thyroid cancer and surrounding invasion, and received palliative resection in 2014 (Fig. 3C and D). Tumor recurrence occurred, and the patient received surgical eradication and regional radiotherapy in September 2015. However, multiple metastases over the neck and anterior chest wall were noted in the subsequent imaging. A biopsy over the neck revealed squamous cell carcinoma, suggesting thyroid transformation. The patient received pembrolizumab (fixed dose of 100 mg every 3 weeks) in August 2016. Restaging based on magnetic resonance imaging (MRI) in November 2016, after 4 courses of treatment, indicated the nodular metastatic lesion over the junction between right lower neck and chest wall was no longer present.

**Figure 4:**
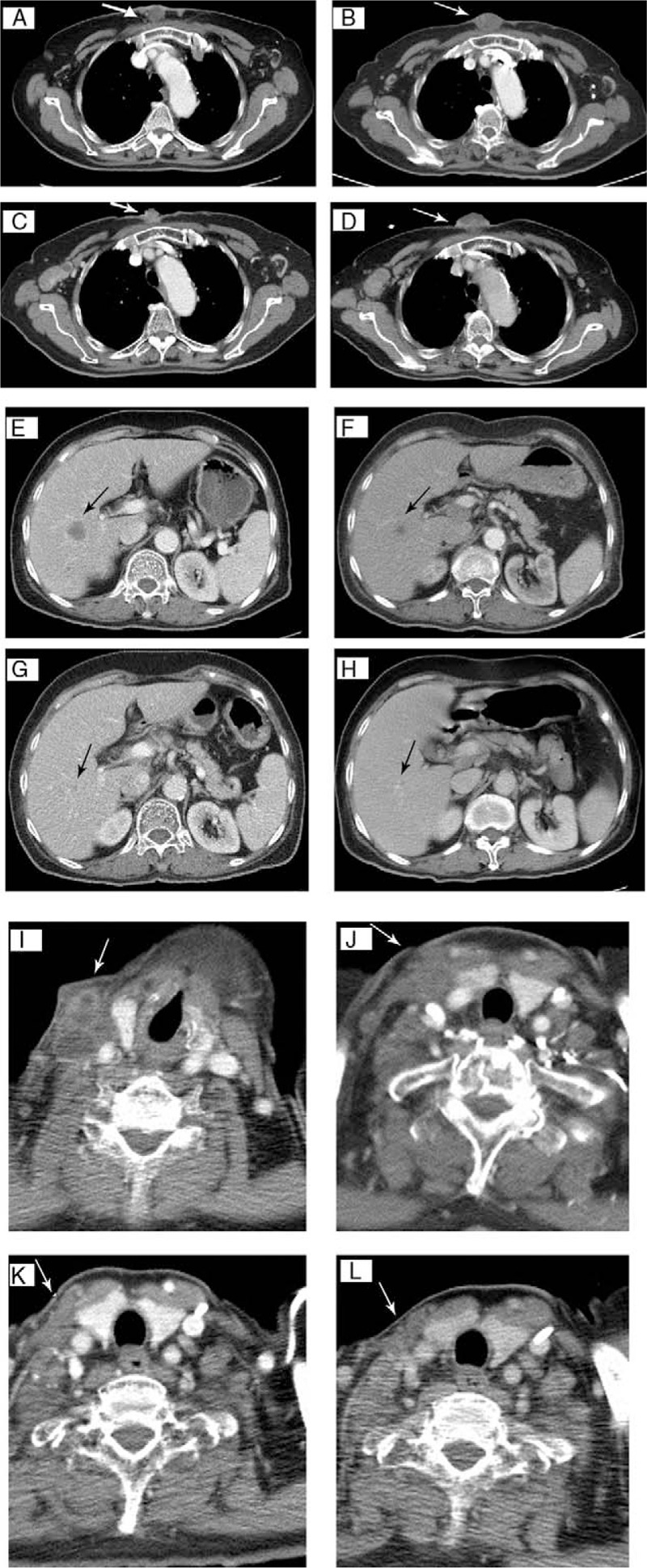
Patient No. 1 had a target lesion in the sternum (white arrows) before cytotoxic chemotherapy treatment (A), disease progression after 3 months (B), partial response after 6 months (C), and progressive disease after 8 months (D). Pembrolizumab led to persistent shrinkage of the liver metastases (black arrows; before treatment [E], 3 months [F], 6 months [G], and 8 months [H] after treatment) and shrinkage of the right neck lymphadenopathies (white arrows; before treatment [I], 3 months [J], 6 months [K], and 8 months [L] after treatment).

Four patients had disease progression, and 1 had continuous progression of the target lesion after shrinkage of other metastases, are all defined as progressed disease. Patient No. 1 was diagnosed with squamous cell carcinoma of unknown primary origin, with a metastatic tumor in the right neck and sternum (Fig. 4). This patient received cytotoxic chemotherapy with avastin, cisplatin, and 5-FU as a first-line treatment in August 2015, but follow-up imaging indicated disease progression. The patient then received gemcitabine, cetuximab, and cisplatin, as second-line and third-line treatments, but disease progression was evident, with metastases in the liver, lung, and sternum. The patient then accepted a fixed dose of pembrolizumab (100 mg every 3 weeks) in February 2016. Follow-up imaging revealed progression of the sternum mass, but regression in the lung, liver, and neck lymphadenopathies after 4 courses of treatment (April 2016). The patient started radiation therapy to treat the sternum mass (4500 cGy in 15 fractions) in May 2016. Pembrolizumab treatment was continued during this time, and a follow-up image in September 2016 indicated a PR, with tumor shrinkage.

### 3.3 Toxicity

**Figure 5:**
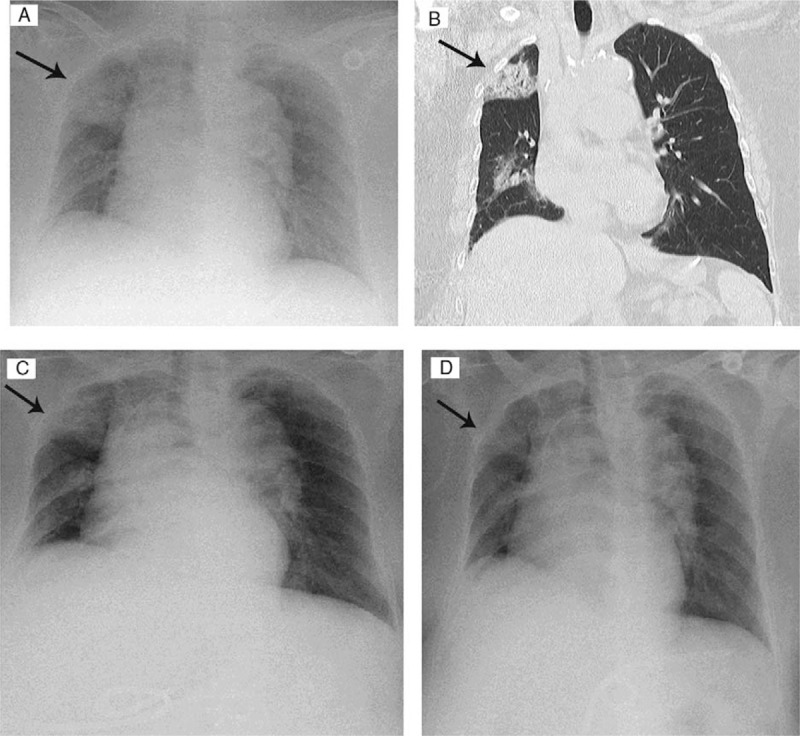
Patient No. 14 had incidentally discovered patchy pneumonia (black arrows) over the right upper lobe (A, chest X-ray; B, chest computed tomography), which had poor response to 5 days of antibiotic treatment (C). Serial images indicate disease resolution following 8 days of methylprednisolone treatment (D).

Treatment-related adverse events occurred in 6 patients (27.27%). One patient developed grade 3 immune therapy-related pneumonitis and led to treatment discontinuation. Another patient developed grade 2 infusion reaction on the 1st administration, and 3 patients had grade 2 subclinical hypothyroidisms (Table 2). Patient No. 14 developed immune-related pneumonitis with grade 3 dyspnea and hypoxia after 3 courses of pembrolizumab (42 days after the first injection) (Fig. 5). This patient initially presented with right upper lobe pneumonia, and did not respond to antibiotics (cefoperazone sodium/sulbactam sodium) after 5 days. Based on suspected immune-related pneumonitis, high-dose intravenous methylprednisolone (2 mg/kg per d) was administered immediately. We quickly tapered the dosage by half every 3 days, due to the rapidly resolving patch based on chest X-rays, and then adjusted to oral prednisone (20 mg/d) for 14 days. The total duration of steroid treatment was 35 days (Fig. 5A–D). Patient No. 11 developed skin rashes with maculopapular eruptions all over the body and itching at 4 h after the initial injection of pembrolizumab. This condition resolved on the next day after antihistamine treatment. Patient No. 2, No. 11, and No. 18 developed subclinical hypothyroidism after at least 3 courses of treatment with pembrolizumab 100 mg. Thyroxine supplement was administered on discovery of subclinical hypothyroidisms with TSH level > 10 mIU/L. (Table 2). No other patients developed severe adverse effects.

**Table 2:**
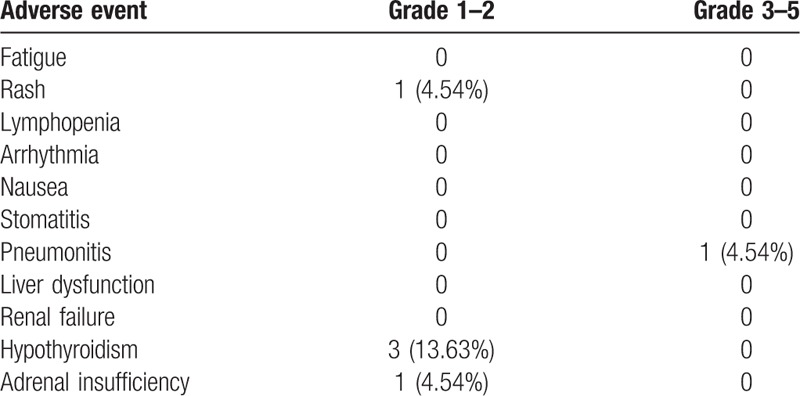
Adverse events in 22 patients in the treated population.

## 4 Discussion

Our study of patients who previously received intensive treatment for R/M HNSCC indicated that pembrolizumab had a good safety profile and promising antitumor activity. Patients with progressive HNSCC after platinum-based regimens have poor prognosis, with a median survival time of only about 3.5 months. Two different antibodies against PD-1 (pembrolizumab and nivolumab) were recently approved as second-line therapies for R/M HNSCC based on 2 pivotal studies.

An open-label, multicenter, phase Ib trial of pembrolizumab for treatment of R/M HNSCC (KEYNOTE-012) enrolled 60 patients who tested positive for the programmed cell death-ligand 1 (PD-L1); 23 (38%) patients were HPV positive and 37 (62%) were HPV negative.^[6]^ The overall rate of drug-related adverse events of any grade was 63% (n = 38), and the most common adverse events were fatigue, pruritus, nausea, decreased appetite, and rash. Ten patients (17%) had grade 3 or 4 drug-related adverse events, the most common of which were increased alanine aminotransferase, increased aspartate aminotransferase, and hyponatremia. The overall response rate was 18%, and the percentage of patients with positive responses was greater among those who were HPV positive (n = 5, 25%) than HPV negative (n = 7, 19%).^[6]^

Another randomized, open-label, phase 3 trial examined patients with R/M HNSCC whose disease had progressed within 6 months after platinum-based chemotherapy (Checkmate-141). Among these previously treated patients, those given nivolumab had improved OS relative to those given single-agent chemotherapy (methotrexate, docetaxel, or cetuximab).^[7]^ In this trial, the nivolumab group had a median OS of 7.5 months, an estimated 1-year survival rate of about 19%, a median PFS of 2.0 months, and a response rate of 13.3%. Treatment-related adverse events of grade 3 or 4 occurred in 13.1% of patients in the nivolumab group. Other trials comparing chemotherapy with checkpoint inhibition antibodies, such as durvalumab (a PD-L1 inhibitor), alone or with tremelimumab (a cytotoxic T-lymphocyte-associated antigen 4 inhibitor, also known as CTLA-4 inhibitor), are still ongoing.^[8]^

The R/M HNSCC patients in our study who received pembrolizumab had a median PFS of 140 days (4.67 months), which is noninferior to the PFS of a previous study (KEYNOTE-012).^[2]^ In addition, our patients had a DCR of 59.1%. Our analysis of the toxicity profile indicated there were very few treatment-related adverse events of grade 3 or 4. Based on previous studies, the incidence and severity of adverse effects were unrelated to pembrolizumab dose.^[9–13]^ It seems that patients had higher response to pembrolizumab if the front-line treatments were ever responsive, although the *P* value is insignificant due to the small number.

HPV status was retrospectively evaluated and only 4 patients had p16 staining data, including 3 patients with p16 negative and 1 patient with p16 positive. Further HPV PCR testing had demonstrated undetectable HPV DNA in the patient with p16 staining positive. The patient received fixed dose of pembrolizumab with 100 mg and follow-up imaging was performed 3 courses later, revealing disease progression. HPV is a major causative factor of HNSCC, especially in cancer arising from oropharyngeal. p-16 protein expression is a biomarker for HPV infection, and it also indicated better prognosis in oropharyngeal HNSCC.^[14]^ However, the role of HPV infection, and the HPV infection correlation with p-16 expression in HNSCCs apart from OPSCC are still not clearly defined.^[15,16]^ Previous studies have revealed that p-16 expression may correlated with PD-L1 expression in nonoropharyngeal HNSCC. However, the p-16 positive patient (Patient No. 9) in our study showed no response to pembrolizumab after 3 courses of treatment. The predictive value of p-16 expression in pembrolizumab treatment response is still be to confirmed.

In our study, 1 patient developed immune-related pneumonitis after 47 days, after 3 courses of pembrolizumab treatment. Pneumonitis is uncommon, but potentially fatal, in checkpoint inhibitor immunotherapy. The median duration of treatment with steroid was 2.8 months (9 days to 19 months), which is variable basing on previous studies. Patients receiving combination of immune checkpoint inhibitors had earlier onset of pneumonitis comparing with those receiving monotherapy (median: 2.7 vs. 4.6 months).^[17,18]^ The median starting dose of prednisone was 50 mg (range: 20–80 mg) and the median corticosteroid treatment duration was 68 days (range: 20–154 days).^[19]^ The most common symptoms were dyspnea (53%) and cough (35%), and one-third of the patients were asymptomatic. Our patient did not initially present with the symptoms mentioned above, and was incidentally discovered to have patchy pneumonia following a general emergency room examination after a falling accident with scalp laceration. Dyspnea and desaturation developed gradually during the course of her hospitalization. The total duration of her treatment was less than reported in other studies, possibly because of early detection.

Another patient developed itchy macular skin rashes at 4 h after receipt of the first pembrolizumab infusion, a response not reported in any previous studies. Skin rash is a common adverse event associated with immune checkpoint antibody therapy, and typically occurs after the second cycle of anti-PD-1 treatment.^[11,20,21]^ Maculopapular rash is the most common type of skin rash,^[21,22]^ and these rashes may be managed with topical or oral corticosteroids, along with oral antipruritic agents (antihistamines).^[17,20,21,23]^ Mild or moderate itching, with or without rash (grade 1 and 2), is generally managed by symptomatic treatment.^[24]^ The symptoms and signs of our patient resolved 4 h after infusion with an antihistamine, suggesting the reaction was related to IgE-related hypersensitivity rather than T-cell associated immune reactions.

Previous studies of the pharmacokinetics of pembrolizumab suggested a dosage of 2 to 10 mg/kg every 3 weeks for a variety of patient subpopulations.^[9,10]^ However, data from KEYNOTE-001 indicated saturation of ex vivo target engagement in blood began at dosages of 1 or more mg/kg every 3 weeks.^[25]^ A mixture model based on initial data from KEYNOTE-001, KEYNOTE-002, and KEYNOTE-006 considered tumor size with a combination of tumor growth and regression terms, as well the fraction of tumor that responded to therapy. The simulated median response rate at week 24 for ipilimumab-naive patients was 44.7% (90% CI: 38.8–49.8) for those given 2 mg/kg every 3 weeks, and was 47.4% (90% CI: 43.7–51.3) for those given 10 mg/kg every 3 weeks. For those with prior receipt of ipilimumab, the response rate at week 24 was 36.9% (90% CI: 32.8–41.3) for those given 2 mg/kg every 3 weeks, and was 38.8% (90% CI: 35.2–42.7) for those given 10 mg/kg every 3 weeks (36.9% vs. 38.8%).^[26]^ The similarity of response rates at 1 mg/kg every 3 weeks and 2 mg/kg every 3 weeks suggests that individuals whose pembrolizumab exposures are reduced by as much as 50% still experience meaningful efficacy.^[26]^

Another preclinical study of mice suggested the lowest dose regimen of pembrolizumab that achieved a maximal response was 2 mg/kg every 3 weeks, with the probability of tumor size reduction at only a slightly lower dose (1 mg/kg every 3 weeks).^[9]^ In our study, 20 patients received a 100 mg fixed dose every 3 weeks, and 2 patients received a 200 fixed dose every 3 weeks. The median dose per patient was 1.625 mg/kg (range: 1.43–2.20), lower than the typical body weight-based dosage (2–10 mg/kg) and lower than the most currently used fixed dosage in other studies (200 mg). However, the response of our patients was still satisfactory. Some case reports also reported that low-dose pembrolizumab led to favorable results. For example, 1 patient with primary resistant Hodgkin lymphoma that was refractory to chemotherapy and autologous peripheral blood stem cell transplantation achieved a very good PR after 4 cycles of pembrolizumab with doses varying from 1 to 2 mg/kg every 3 weeks.^[27]^ Another case report described 2 patients with classical Hodgkin lymphoma who achieved complete remission and near-complete remission after 4 and 6 courses of pembrolizumab with dosages of 2 mg/kg every 3 weeks.^[28]^

Our patients also had nonconcordant changes of metastatic lesions and target lesions, possibly because varying expression of PD-1 led to different effects following the same treatment.^[7]^ In addition, the role of PDL-2, another ligand of PD-1, is still poorly understood. The KEYNOTE-012 trial showed discordance between PDL-1 and PDL-2 expression in a subpopulation of patients, and the researchers suggested that PDL-2 expression is associated with a higher overall response rate after adjusting for PDL-1 expression.^[6]^

There are still some limitations of our study. First, it is retrospectively designed so the selection bias is inevitable. Second, the sample size is relatively small so the correlation analysis is difficult.

In conclusion, our study of patients with R/M HNSCC indicated that pembrolizumab prolonged survival and is associated with a low incidence of toxic effects relative to conventional systemic chemotherapy.
